# Longitudinal plasma proteome profiling after allogeneic hematopoietic stem cell transplantation: heterogeneous development and associations with acute GvHD

**DOI:** 10.3389/fimmu.2026.1792903

**Published:** 2026-09-28

**Authors:** Iris C. Kreft, Myrddin W. Verheij, Maartje van den Biggelaar, Ingrid Bulder, Eva R. Smit, Carmen van der Zwaan, Floris P. J. van Alphen, Sacha S. Zeerleder, Mette D. Hazenberg, Pieter Langerhorst, Carlijn Voermans

**Affiliations:** 1 Sanquin Research and Landsteiner Laboratory, Amsterdam, Netherlands; 2 Department of Hematology, Division of Internal Medicine, Kantonsspital Lucerne, Lucerne and University of Berne, Berne, Switzerland; 3 Cancer Center Amsterdam and Amsterdam Infection and Immunity Institute, Amsterdam University Medical Center, Amsterdam, Netherlands; 4 Department of Hematology, Amsterdam University Medical Center, University of Amsterdam, Amsterdam, Netherlands

**Keywords:** allogeneic hematopoietic stem cell transplantation, graft versus host disease (GVHD), immunoglobulins, mass spectrometry, proteomics

## Abstract

Allogeneic hematopoietic stem cell transplantation (HSCT) offers a potential cure for malignant and non-malignant hematopoietic diseases, but the development of acute graft-versus-host disease (GvHD) caused by donor immune cell-mediated inflammation and destruction of host tissues poses a significant challenge. It remains difficult to predict whether and when patients will develop acute GvHD. Here, we used mass-spectrometry (MS)-based proteomics to identify plasma proteins associated with allogenic HSCT and acute GvHD development. In this study, we analyzed longitudinal plasma samples at baseline (pre-HSCT), 1, 3, 6, and 12 months post-HSCT from 10 allogeneic HSCT recipients, 7 of whom developed acute GvHD, and 5 healthy donors. As expected, significant differences between the plasma proteomes of allogeneic HSCT recipients and healthy donors were found. The most pronounced differences were observed 3 months post-HSCT, with four proteins (C4A, FTL, GNPTG, S100A9) that were altered before allogeneic HSCT and remained altered up to 12 months post-HSCT compared to healthy donors. Three other proteins (VWF, RNASE4, B2M) showed alterations only after transplantation. Moreover, co-expression analysis revealed an association between inflammation-related proteins (ITIH3, ITLN1, COLEC11, SERPING1, SERPIND1 and IgG) and acute GvHD grade. Together, we demonstrate how plasma proteomic profiling can support the monitoring of acute GvHD in allogeneic HSCT recipients.

## Introduction

Allogeneic hematopoietic stem cell transplantation (HSCT) is often the only curative option for patients with hematological malignancies such as acute myeloid leukemia (AML) or non-malignant blood disorders such as sickle cell disease (SCD) (1–3). Currently however, the application of allogeneic HSCT in patients is limited by acute graft-versus-host disease (GvHD), a common and potentially severe complication in which tissues and organs of the patient are targeted and destroyed by donor immune cells present in the transplanted graft (4). Studies have shown that the development of acute GvHD is largely dependent on tissue damage that is induced by the conditioning regimen that patients receive before transplantation and by infections (5, 6). As such, many studies have tried to unravel the specific factors involved in the process, but thus far it remains very difficult to predict whether a patient will develop acute GvHD after their transplantation or not (7–9). Two biomarkers, ST2 and Reg3α, have emerged as important indicators of graft-versus-host disease development and severity, with Reg3α identified through proteomics-based discovery approaches and ST2 through targeted biomarker analyses. Reg3α is primarily associated with gastrointestinal GvHD and intestinal epithelial damage (10), whereas elevated ST2 levels have been linked to steroid-refractory disease and increased non-relapse mortality (11). The work of the Paczesny lab (12) has shown that a comprehensive panel of 6 markers is able to predict mortality and treatment responsiveness in patients when measured upon GvHD onset. In addition, the MAGIC biomarker panel, measured after initiation of GvHD therapy, is able to predict the long-term outcome in patients with steroid-resistant GvHD much more accurately than was previously possible making use of clinical criteria alone (13). Prediction of GvHD development however remains difficult, which is why the discovery and validation of robust biomarkers that can predict or early-detect acute GvHD is still critical to improve the management and prognosis of allogeneic HSCT recipients.

High-throughput plasma proteomics using mass spectrometry (MS) enables the unbiased analysis of several hundreds of proteins, offering a comprehensive view of the plasma proteome and potentially uncovering protein markers and molecular pathway changes associated with disease (14–16). In this pilot study, we aimed to analyze the differences in the plasma protein profiles of allogeneic HSCT patients with and without acute GvHD and compared their plasma proteomes to those from healthy donors. We quantified plasma proteomic changes associated with allogeneic HSCT and acute GvHD development using MS-based proteomics in longitudinal samples collected from 10 allogeneic HSCT recipients at consecutive standardized time points before and after transplantation and correlated these with clinical parameters.

## Methods

### Study participants and sample collection

For this study we made use of prospectively bio-banked plasma samples, the collection of which was approved by the Medical Ethics Committee of the Amsterdam UMC, Amsterdam, The Netherlands. For this biobank EDTA plasma is collected at baseline (0–7 days pre-transplant), and 1, 3, 6, and 12 months after transplantation. We selected n=10 allogeneic HCT recipients from the biobank who were all treated with a reduced intensity conditioning regimen (RIST, busulfan/fludarabine or cyclophosphamide/fludarabine) prior to transplantation. All patients participated in the HOVON 96 clinical trial, which compared shorter and longer duration GvHD prophylaxis with cyclosporin A and mycophenolic acid (arms 1 and 2) to GvHD prophylaxis with post-transplantation cyclophosphamide (arm 3). Nine out of 10 patients were part of arms 1 and 2 of the trial and 1 patient was part of arm 3 (17). The included patients with acute GvHD were diagnosed in the first 100 days after transplantation (**Supplementary Figure 1A**). In addition, plasma samples were collected from five healthy adults in accordance with Dutch regulations and Sanquin Ethical Advisory Board approval, following the Declaration of Helsinki.

For each patient, clinical parameters including platelet-, leukocyte-, and neutrophil counts, hemoglobin (Hb), creatinine, bilirubin, aspartate aminotransferase (AST), alanine aminotransferase (ALT), gamma-glutamyl transferase (GGT), alkaline phosphatase (ALP), and lactate dehydrogenase (LDH) levels were also acquired. Nucleosomes (18) (AU/ml), hemopexin (19) (µg/ml), heme oxygenase-1 (19) (HO-1, pg/ml), the human neutrophil elastase-α1-antitrypsin complex (HNE-a1AT, ng/ml), C3b/c15 (nM), C4b/c (nM) (20), α1-antitrypsin (a1AT, µg/ml), haptoglobin (µg/ml) (19) and the S100A8/A9 complex (21, 22) (calprotectin, µg/ml) were determined by ELISA as described previously or as detailed in the supplemental methods.

### Proteomics analysis

Plasma samples were prepared for MS-based proteomic analysis as described previously (**Supplementary Figure 1B**) (23). Peptides were separated using C18 reverse phase liquid chromatography (24) coupled on line to an Orbitrap Fusion Lumos Tribrid mass spectrometer (Thermo-Fisher Scientific), which was operated in data-independent acquisition (DIA) mode. In DIA, an MS1 scan was conducted in the Orbitrap analyzer, from 400 to 1000 m/z, at 60K resolution (AGC target of 4x10^5^). Subsequently, 75 DIA segments were acquired at 15K resolution (AGC target 4 x10^5^) with a maximal injection time of 23 ms and an isolation width of 12 m/z. HCD fragmentation with normalized collision energy of 33% was used, with a default charge state of 3 for MS2. Spectra were recorded in centroid mode, with parallelizable time enabled. Raw data were analyzed by DIA-NN software (version 1.8) and searched against the human proteome (Swiss-Prot Database, 20,361 entries as of April 11, 2022) using a predicted library-free search. Parameters included protein inference based on FASTA protein names, robust LC quantification mode (high accuracy), match between runs enabled, and no shared spectra, without heuristic protein inference strategy.

### Data analysis

The output files were processed in Rstudio, allocating a seed number to allow for reanalysis. Only protein groups identified by at least two distinct precursors were included for quantification. Across a large dynamic range (**Supplementary Figures 1C, D**), 493 proteins were identified in >70% of the samples and used for further analysis (**Supplementary Table 1**). Missing values were imputed by normal distribution (width = 0.3, shift = 1.8). Differential protein abundances were determined by moderated t-testing between healthy controls and each HSCT-timepoint (limma-package (25)). Proteins were considered significantly different if P-adj <0.05 (method = Benjamini-Hochberg) and absolute log2 fold change >1 of intensities was present. Inter- and intra-patient developments after allogeneic HSCT were determined by calculating the coefficients of variation (CVs) of non-log2 transformed protein intensities versus the log2-fold change difference from baseline for each allogeneic HSCT recipient. For immunoglobulin (Ig) peptide analysis, we extracted intensities from peptides specific to Ig classes/type excluding those associated with known genetic variations as documented in the IMGT database (26). These peptides were then cross-referenced with peptides derived from our experimental results. In instances where more than two peptides were associated with a single Ig type, the peptide levels were averaged to consolidate them into a single level for each Ig type (**Supplementary Table 1**). Co-expression analysis was performed on laboratory parameters and protein levels using spearman correlation coefficient calculated with the Hmisc package (27). To establish the association of proteins with acute GvHD, we conducted two correlation analyses. In the first approach, we assigned the categorical acute GvHD grade to each time point for an individual; for instance, if patient X had acute GvHD grade 3, all samples were annotated grade 3. In the second approach, we annotated only the time points corresponding to the diagnosis of acute GvHD and the subsequent time points; for example, if patient X was diagnosed at 3 months after HSCT with grade 3, time points 3, 6, and 12 months were annotated with grade 3.

## Results

To investigate the changes in the plasma proteome after allogeneic HSCT, we compared plasma samples of pre- and post-transplantation time points with those of healthy adults. A total of 47 proteins were found to be significantly different between allogeneic HSCT recipients and healthy donors (**Figure 1A**; **Supplementary Figure 2**), with the highest number of differentially abundant proteins (n=39) at 3 months post-HSCT (**Figure 1B**). As the 3-month time point represents the earliest moment at which all patients (7 out of 10) in the study had developed acute GvHD, this suggests that acute GvHD influences the plasma proteome in the context of allogeneic HSCT. Changes were predominantly characterized by a decreased abundance of immunoglobulins and an increased abundance of proteins involved in iron metabolism (ferritin light chain [FTL], hepcidin [HAMP]), inflammation (S100 calcium-binding protein A8 [S100A8], S100 calcium-binding protein A9 [S100A9]), complement (complement component C4A [C4A]) and a range of other proteins (**Supplementary Table 1**).

**Figure 1:**
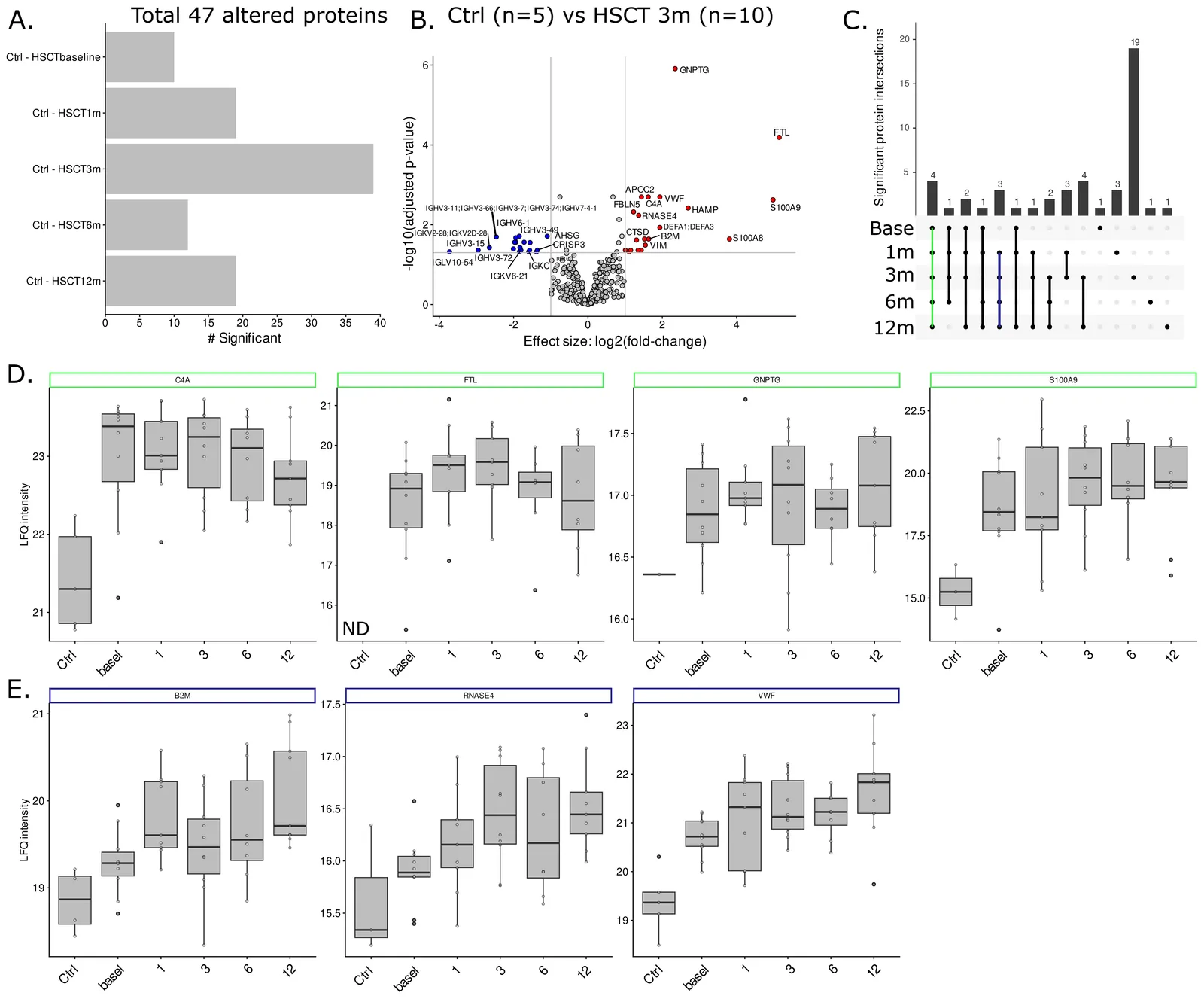
Mass spectrometry-based profiling reveals significantly altered plasma proteins in allogeneic HSCT recipients compared to healthy donors. **(A)** Number of significantly altered proteins between the plasma proteomes at timepoints pre-HSCT, post-HSCT (1-,3-6-,12- months) and those of healthy donors (n=5). Individual comparisons are shown as separate volcano plots in **Supplementary Figure 2**. **(B)** Volcano plot comparing the plasma proteomes of allogeneic HSCT recipients at 3 months post-HSCT (n=10) to those of the healthy donors (n=5). Proteins with a significantly higher abundance in allogeneic HSCT recipients are highlighted in red and proteins with significantly decreased abundance in blue. The log_10_ fold-change in protein levels is represented on the x-axis and the -log_10_ adjusted P-value on the y-axis. **(C)** Overlap and unique counts of significantly altered proteins identified in comparisons between healthy controls and HSCT recipients at each time point. Proteins consistently altered across all comparisons are indicated (green), while proteins specifically altered in post-HSCT time points are indicated (blue). **(D)** Label-free quantification (LFQ) intensities of four proteins that were consistently and significantly altered between healthy controls and allogeneic HSCT recipients across all time points, as identified from the overlap analysis shown in **(C)** Data represent raw (non-imputed) values.; C4A: complement 4A; FTL: ferritin light chain; GNPTG: n-acetylglucosamine-1-phosphotransferase subunit gamma; S100A9: Protein S100-A9. **(E)** LFQ intensities of three proteins that were significantly altered between healthy controls and allogeneic HSCT recipients specifically at post-HSCT time points, as identified from the overlap analysis in **(C)** Data represent raw (non-imputed) values. VWF: von Willebrand factor; RNASE4: Ribonuclease 4; B2M: Beta-2-microglobulin.

To assess if common proteomic signatures exist among different time points, we investigated similarities and uniqueness in proteins between baseline, 1, 3, 6 and 12 months post-HSCT (**Figure 1C**). Four proteins (C4A, FTL, S100A9 and N-acetylglucosamine-1-phosphate transferase, gamma subunit [GNPTG]) demonstrated increased abundance at all pre- and post-HSCT time points compared to healthy controls (**Figure 1D**). Three additional plasma proteins (von Willebrand factor [VWF], Ribonuclease 4 [RNASE4] and β2 microglobulin [B2M]) were significantly increased in abundance after allogeneic HSCT (1, 3, 6 and 12 months) (**Figure 1E**), of which VWF was also increased at baseline, albeit not significant.

To distinguish longitudinal changes within individuals from inter-individual variability, we calculated the log fold change (LFC) between baseline and each subsequent time point, reflecting the magnitude of change over time, and the coefficient of variation (CV) across all time points, capturing variability between individuals. Most proteins (52%) showed high inter-individual variability (CV > 30%) (**Supplementary Table 1**; **Figure 2A**). In contrast, only a small proportion of proteins showed substantial longitudinal changes, with 3.5 and 7.8% displaying an LFC < −1 or LFC > 1, respectively. Together, these findings indicate that inter-individual variability exceeds longitudinal changes within individuals. Proteins related to inflammation and immunoglobulins displayed significant variation and log-fold-change over time, shown by significant variability in LFQ-intensity levels of IGHD, SAA1, and S100A8/A9 (**Figure 2B**), and immunoglobulin-specific peptide profiles (**Figure 2C**).

**Figure 2:**
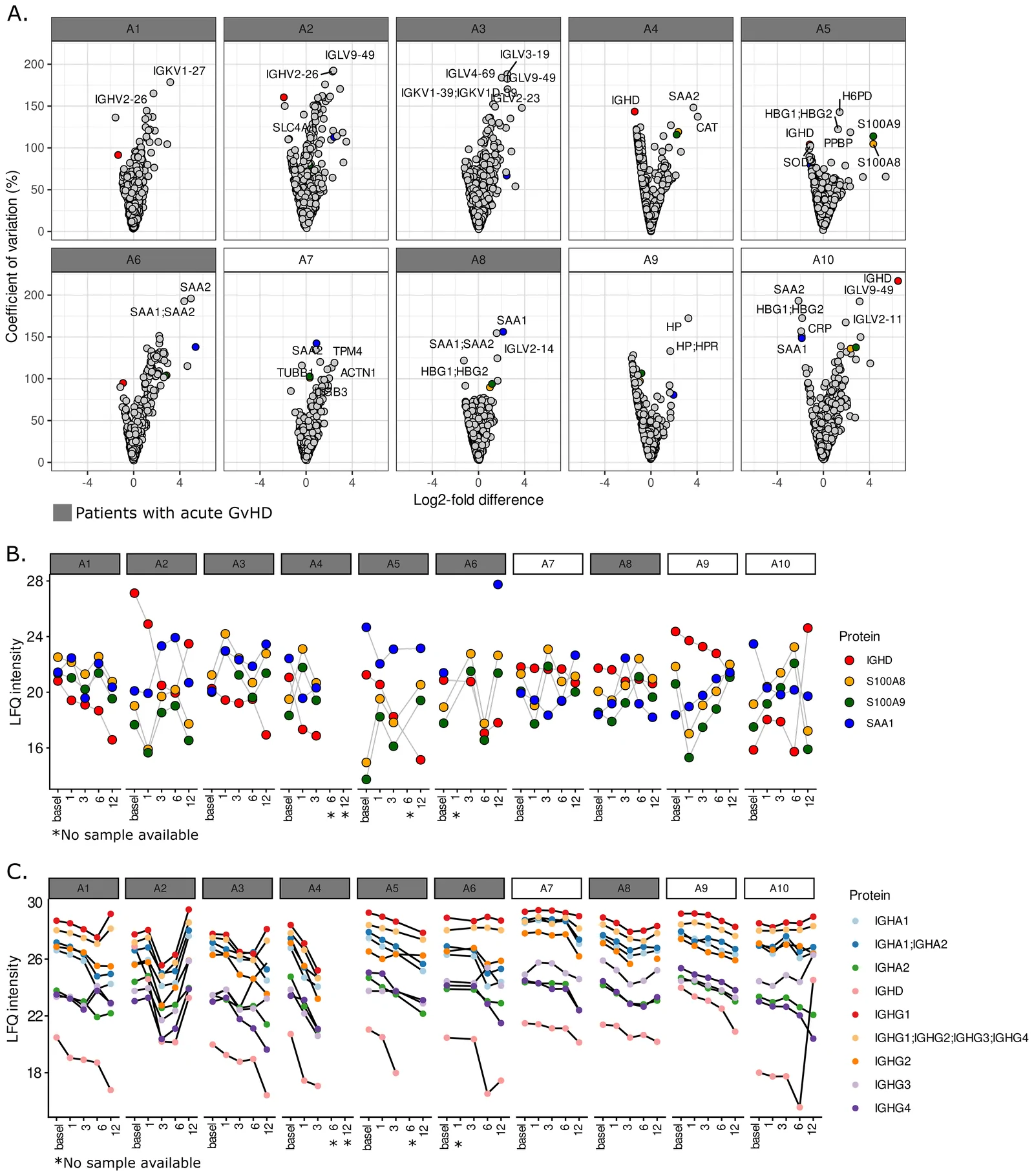
Longitudinal variation in plasma proteome in allogeneic HSCT recipients. **(A)** Plot for each allogeneic HSCT recipient representing the plasma protein log2-fold difference from pre-HSCT to post-HSCT versus percentage of coefficients of variation (% CV). Proteins with CV>100 and LFC>1 are annotated. Each patient is indicated on top, with in grey patients with acute GvHD. **(B)** LFQ intensities of seven proteins plotted after allogeneic HSCT for all 10 recipients. These proteins are stable over time within individuals but strongly vary between individuals. IGHD: Immunoglobulin heavy constant delta; SAA1: Serum amyloid A protein 1; S100A8: S100 calcium-binding protein A8; S100A9: S100 calcium-binding protein A9. **(C)** LFQ intensities of immunoglobulin-specific peptides. Each patient is indicated on top, with in grey patients with acute GvHD. IGHA1: Immunoglobulin heavy constant alpha 1; IGHA2: immunoglobulin heavy constant alpha 2; IGHD: immunoglobulin heavy constant delta; IGHG1: immunoglobulin heavy constant gamma 1; IGHG2: immunoglobulin heavy constant gamma 2; IGHG3: immunoglobulin heavy constant gamma 3; IGHG4: immunoglobulin heavy constant gamma 4.

To relate our results to the clinical presentation of patients, we performed a global correlation analysis between clinical laboratory values (blood counts and liver enzymes), additional laboratory values (e.g., plasma nucleosomes, calprotectin), acute GvHD grade and protein LFQ-intensities from all available samples of allogeneic HSCT recipients (**Supplementary Table 1**). We observed a positive correlation of the liver enzyme aspartate aminotransferase (AST) with polymeric immunoglobulin receptor (PIGR; r=0.62, p=3.99×10^-6^) and aminopeptidase N (ANPEP; r=0.54, p=9.42×10^-5^) (**Figure 3A**) and of the liver enzyme alanine aminotransferase (ALT) with fructose-bisphosphate aldolase (ALDOB; r=0.67, p=7.78×10^-7^), fumarylacetoacetate hydrolase (FAH; r=0.61, p=1.95×10^-5^) and PIGR (r=0.52, p=2.17×10^-4^), (**Figure 3B**). Moreover, we observed a moderate positive correlation (r=0.43 S100A8, r=0.44 S100A9) between the quantified levels of calprotectin determined through ELISA (measured as the complex of S100A8:S100A9) and MS (measured as individual proteins) (**Figure 3C**). In addition, platelet counts showed a strong correlation with thrombospondin-1 (THBS1, r=0.76, p=1.07×10^-9^), platelet basic protein (PPBP, r=0.75, p=1.81 ×10^-9^), and platelet factor 4 (PF4, r=0.66, p=1.06×10^-6^) (**Figure 3D**). When assessing the grade of acute GvHD and taking all time points into account, Inter-alpha-trypsin inhibitor heavy chain H3 (ITIH3, r=0.56, p=4.13×10^-5^) and Intelectin-1 (ITLN1, r=0.57, p=5.16x10^-5^) correlated positively and Collectin-11 (COLEC11, r=-0.71, p=2.71x10^-8^) correlated negatively with the grade of acute GvHD (**Figure 3E**). When performing the same analysis, but only including the time points at which patients had been diagnosed with acute GvHD, we found the acute phase protein C1-inhibitor (SERPING1, r=0.63, p=2.14x10^-6^) to be positively correlated with the grade of acute GvHD (**Figure 3F**). Lastly, we observed a negative correlation between the abundance of IgG and the grade of acute GvHD in both analyses.

**Figure 3:**
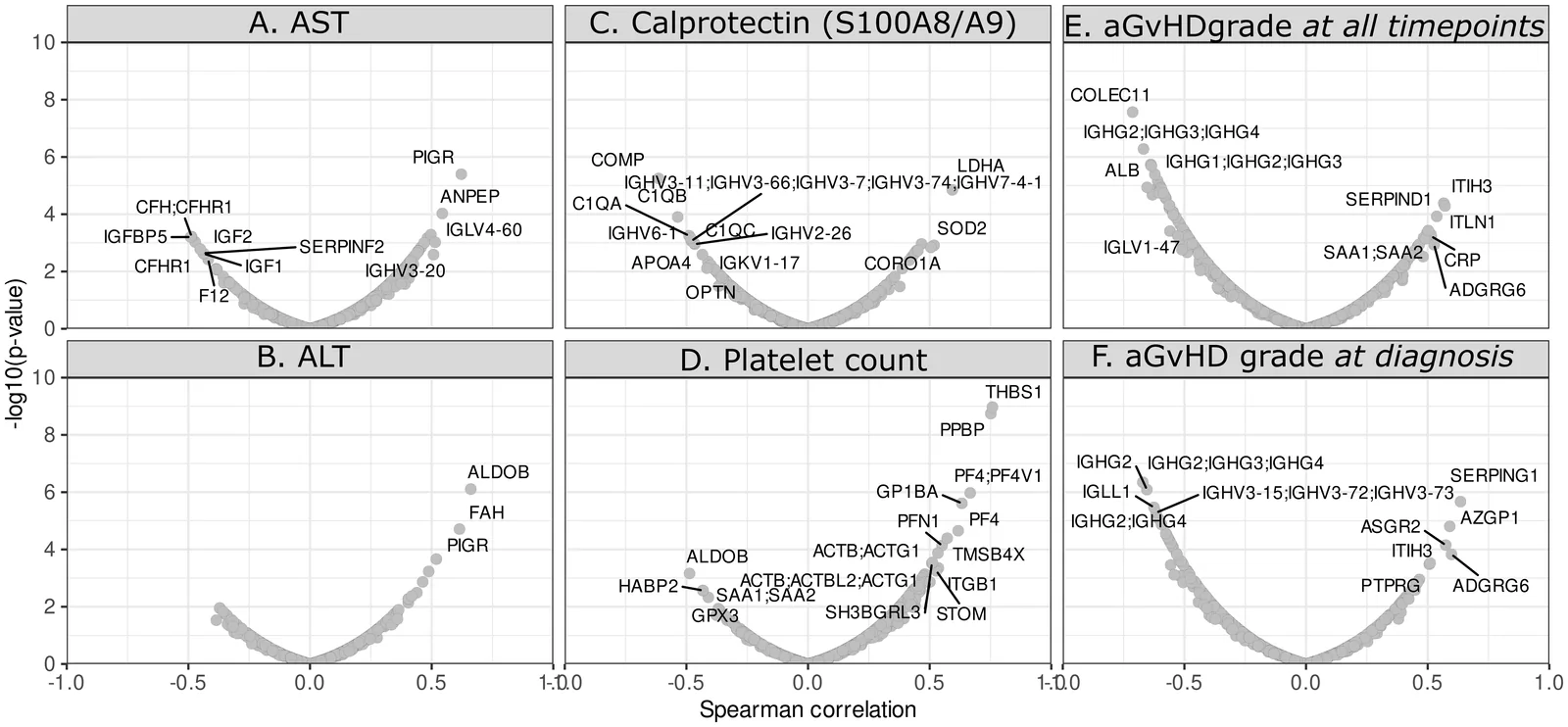
Correlation analysis reveals strong correlation between proteins measured with mass spectrometry and laboratory measurements and indicates an association with acute GvHD. Each plot shows Spearman correlation coefficients (x-axis) versus −log10 (p-values) (y-axis). Selected proteins with the strongest associations are annotated. Panels represent correlations with: **(A)** AST **(B)** ALT **(C)** Calprotectin (complex of S100A8/A9). **(D)** platelet counts. **(E)** Acute GvHD grade at all time points. **(F)** Acute GvHD grade from diagnosis onwards (median 30 [28-62] days post-HSCT).

## Discussion

Outcomes of allogeneic HSCT in patients with hematological malignancies and non-malignant blood disorders are negatively affected by the development of acute GvHD. Unfortunately, it remains difficult to accurately predict whether patients will develop acute GvHD due to a lack of reliable biomarkers. A recent study in patients with chronic GvHD identified the protein Dickkopf-related protein 3 (DKK3) as a novel biomarker for both sclerotic and non-sclerotic chronic GvHD (28), showing the potential of MS-based proteomics for the discovery of new disease-related biomarkers. In this pilot study, we analyzed the plasma proteome of 10 allogeneic HSCT recipients at different time points before (baseline, during HSCT conditioning ranging from day –7 to day 0) and after (1, 3, 6 and 12 months) allogeneic HSCT and of 5 healthy donors using an MS-based proteomics approach. We aimed to improve our understanding of the changes that occur in the plasma proteome after allogeneic HSCT and in relation to acute GvHD development.

The proteomic profiles of allogeneic HSCT recipients diverged from those of healthy donors. At baseline, before transplantation, allogeneic HSCT recipients demonstrated increased abundance of proteins involved in iron regulation such as ferritin light chain (FTL) and hepcidin (HAMP) and inflammation such as C4A. HAMP has also been shown to be increased in conditions characterized by inflammation (29, 30). This is to be expected, as all allogeneic HSCT recipients included in the study had an underlying malignancy and received a conditioning regimen that consisted of chemotherapy and/or radiotherapy, which has been shown to induce tissue damage and inflammation (4). The differences in plasma proteome between allogeneic HSCT recipients and healthy donors became more apparent at the time points after allogeneic HSCT, with a steadily increasing abundance of proteins such as B2M and VWF. The largest number of significantly altered proteins was found 3 months after allogeneic HSCT. As the 3-month time point represents the earliest moment at which all patients (7 out of 10) in the study had developed acute GvHD, this suggests that acute GvHD further influences the plasma proteome in the context of allogeneic HSCT.

We then examined the variability in protein profiles in the 10 allogeneic HSCT recipients over time and found that, compared to baseline, abundance of the acute phase proteins SAA1 and SAA2 as well as proteins related to platelets and iron regulation were increased after allogeneic HSCT. Furthermore, we found that a significant portion of the variability seen in patients after allogeneic HSCT could be contributed to immunoglobulins. This is not surprising, as the humoral immunity needs to recover after being ablated by the conditioning regimen. It has been shown that recovery of the (donor-derived) B cell compartment in patients after allogeneic HSCT is slow and can take up to 5 years (31, 32). Moreover, decreased levels of circulating immunoglobulins, complexity of gene rearrangement patterns and impaired class switching are often seen in patients after allogeneic HSCT, rendering them more susceptible to bacterial and viral infections (33). Our data show that reconstitution of humoral immunity seems to be different for each patient.

When assessing the grade of acute GvHD and taking all time points into account, ITIH3 and ITLN1 correlated positively and COLEC11 correlated negatively with the grade of acute GvHD. ITIH3 and ITLN1 have both been implicated to play a role in the inflammatory response (34, 35), while COLEC11 encodes the protein CL-K1, which is an important player in the lectin pathway of complement (36). When performing the same analysis, but only including the time points at which patients were already diagnosed with acute GvHD, we found the acute phase protein C1-inhibitor (C1-Inh) (encoded by the SERPING1 gene) to be positively correlated with the grade of acute GvHD. C1-Inh plays a pivotal role in regulation of both the complement system and the contact activation system (involving coagulation factors XI and XII, prekallikrein and high molecular weight kininogen) (37). Lastly, we observed a negative correlation between the abundance of IgG and the grade of acute GvHD in both analyses. Hypogammaglobulinemia is a common occurrence in patients after allogeneic HSCT, and low IgG levels (<400 mg/dL) have been shown to correlate with increased transplant-related mortality (38). Importantly, acute GvHD was found to be one of the significant factors contributing to low levels of IgG after allogeneic HSCT (39, 40). Interestingly, our observations did not match the biomarkers used in the panel developed by the Paczesny lab and in the MAGIC study (e.g. Reg3α and ST2) (12, 13). However, these were large-scale studies focused on outcome predictions in patients after GvHD diagnosis and for specific subsets of disease (e.g. gastrointestinal GvHD), whereas we performed a pilot study, focused on prediction of outcome before onset of acute GvHD. Taken together, our data derived from this small cohort of allogeneic HSCT recipients confirm the importance of the inflammatory response in acute GvHD pathogenesis.

Finally, our global correlation analysis shows that the MS-based protein quantification agrees with clinical measurements, providing a positive control for our workflow. In addition, we found strong correlations between platelet counts in patients and platelet-related proteins measured by MS, further showing the robustness of the technique.

Taken together, we show that the plasma proteome of patients undergoing allogeneic HSCT is different from healthy donors prior to transplantation, and that these differences become more apparent after transplantation. Notably, we found the abundance of several proteins related to iron regulation and inflammation to be persistently altered up to 12 months after allogeneic HSCT.

It is important to note that this study was constrained by small patient and healthy donor sample sizes, diminishing the statistical power available for conducting comparative analyses. Moreover, the healthy donor group was comprised of only females. Larger studies with an even distribution of males and females are needed to determine the effects of sex as a biological variable. It is also important to note that we did not take immune reconstitution and the effects of potential chronic GvHD into account in our analyses, as these were beyond the scope of this pilot study. Nevertheless, we identified several plasma proteins associated with inflammation that correlated with the occurrence of acute GvHD. Given that the MS-based proteomics technique is non-invasive and only requires ~20 µl of plasma to comprehensively analyze protein profiles in a high-throughput manner, these findings highlight the potential of MS-based proteomics and the opportunities for future investigations involving larger patient cohorts.

## Data Availability

The RAW and Search Files are stored in the PRIDE repository with number PXD04798.
