# Supplementary material for: Longitudinal plasma proteome profiling after allogeneic hematopoietic stem cell transplantation: heterogeneous development and associations with acute GvHD
Source: Front Immunol. 2026 Sep 28;17:1792903. doi: 10.3389/fimmu.2026.1792903 (PMC13654744; doi:10.3389/fimmu.2026.1792903)
Supplement: Supplementary file 1 [file SupplementaryFile1.docx]

**Supplemental Figures**

**Supplemental Figure S1: Patient characteristics and mass spectrometry-based plasma profiling**

**A.** Table of characteristics of patients (n=10) and healthy donors (n=5). Seven out of 10 patients developed acute GvHD, of which 5 patients developed both acute and chronic GvHD (graded using the classic Glucksberg-Seattle criteria). RIST = reduced-intensity hematopoietic stem cell transplantation, aGvHD = acute graft-versus-host disease, HSCT = hematopoietic stem cell transplantation, - = not applicable).

**B.** Overview of the study design, blood samples from allogeneic HSCT recipients (in blue) drawn at multiple time points; pre-transplant (baseline), 1, 3, 6, 12-months (m) post-transplant and 1 single time point for healthy donors (in purple) were collected. Plasma was obtained by centrifugation of whole blood at 1000*g* for 5 min, and subsequent proteolytic digestion yielded peptides. Peptides were purified using C18 cartridges on the AssayMAP Bravo platform (Agilent technologies) and analysed by Liquid chromatography tandem mass spectrometry (LC-MS/MS) in data-independent acquisition mode. In DIA, an MS1 scan was conducted in the Orbitrap analyzer, from 400 to 1000 m/z, at 60K resolution (AGC target of 4x10^5^). Subsequently, 75 DIA segments were acquired at 15K resolution (AGC target 4 x10^5^) with a maximal injection time of 23 ms and an isolation width of 12 m/z. HCD fragmentation with normalized collision energy of 33% was used, with a default charge state of 3 for MS2. Spectra were recorded in centroid mode, with parallelizable time enabled. Raw data were analyzed using DIA-NN (version 1.8) software, searching against the human proteome (Swiss-Prot Database, 20,361 entries as of April 11, 2022) via a predicted library-free search. Parameters included protein inference based on FASTA protein names, robust LC quantification mode (high accuracy), match between runs enabled, and no shared spectra, without heuristic protein inference strategy. The output files were processed in RStudio, where only protein groups matched to a minimum of two distinct precursors were included for quantification.

**C.** Quantitative LFQ-intensities of 542 proteins ranked according to their abundance, covering more than five orders of magnitude of mass spectrometer signal with several proteins exemplified over the abundance range (green dots). Protein levels were log2-transformed and quantified if detected in ≥70% of samples. Missing values were imputed using a normal distribution (width = 0.3; shift = 1.8).

**D.** Box plots illustrate the protein count in plasma samples from allogeneic HSCT recipients (blue) containing 542 [480 – 659] proteins and healthy donors (purple) with 530 [496 – 517] proteins.

**Supplemental Figure S2. Comparison between the plasma proteome of healthy controls and HSCT-recipients at different time points.** Volcano plots of expression changes in allogeneic HSCT recipients pre- and 1, 3, 6, 12 months post-HSCT compared to healthy donors (CTRL). Red dots indicate upregulated proteins, blue dots indicate downregulated proteins. Protein significance was determined with moderated t-tests using the limma-package20 and Benjamini-Hochberg correction. Proteins were considered significant and relevant with P-adj <0.05 and an absolute log2 fold change >1**.**
